# Exploring the Immunological Role of the Microbial Composition of the Appendix and the Associated Risks of Appendectomies

**DOI:** 10.3390/jpm15030112

**Published:** 2025-03-14

**Authors:** Tarequl Islam, Md Shahjalal Sagor, Noshin Tabassum Tamanna, Md Kamrul Islam Bappy, Danishuddin, Md Azizul Haque, Maximilian Lackner

**Affiliations:** 1 Department of Microbiology, Noakhali Science and Technology University, Noakhali 3814, Bangladesh; tarequembg@gmail.com (T.I.); kamrul0509@student.nstu.edu.bd (M.K.I.B.); 2 Department of Microbiology, Jagannath University, Dhaka 1100, Bangladesh; sagor@squaregroup.com; 3 Department of Pharmacy, Noakhali Science and Technology University, Noakhali 3814, Bangladesh; tamannanoshin@gmail.com; 4 Department of Biotechnology, Yeungnam University, Gyeongsan 38541, Republic of Korea; danish23@yu.ac.kr; 5 Department of Industrial Engineering, University of Applied Sciences Technikum Wien, Hoechstaedtplatz 6, 1200 Vienna, Austria

**Keywords:** appendectomy, appendix microbiome, colorectal cancer, dysbiosis, gut microbiome, immune component

## Abstract

The appendix, an integral part of the large intestine, may serve two purposes. First of all, it is a concentration of lymphoid tissue that resembles Peyer’s patches. It is also the main location in the body for the creation of immunoglobulin A (IgA), which is essential for controlling intestinal flora’s density and quality. Second, the appendix constitutes a special place for commensal bacteria in the body because of its location and form. Inflammation of the appendix, brought on by a variety of infectious agents, including bacteria, viruses, or parasites, is known as appendicitis. According to a number of studies, the consequences of appendectomies may be more subtle, and may relate to the emergence of heart disease, inflammatory bowel disease (IBD), and Parkinson’s disease (PD), among other unexpected illnesses. A poorer prognosis for recurrent *Clostridium difficile* infection is also predicted by the absence of an appendix. Appendectomies result in gut dysbiosis, which consequently causes different disease outcomes. In this review, we compared the compositional differences between the appendix and gut microbiome, the immunological role of appendix and appendix microbiome (AM), and discussed how appendectomy is linked to different disease consequences.

## 1. Introduction

The human appendix, an anatomical structure characterized by its slender, tubular projection from the cecum situated in the lower right quadrant of the abdomen, has remained a focal point of scientific inquiry [1]. Frequently categorized as a trivial organ, the appendix continues to captivate scientific interest due to its intriguing presence within human anatomy [1,2]. Morphologically, the appendix typically measures approximately 9 cm in length, exhibiting variations ranging from 5 to 35 cm, with a diameter averaging around 6 mm [2,3].

The anatomical positioning of the appendix is notably variable, impacting both diagnostic procedures and surgical intervention. The most common anatomical orientation of the appendix is retrocecal, located posterior to the cecum, accounting for approximately 65–70% of all cases. Alternatively, in about 25–30% of individuals, the appendix descends into the pelvic region, known as the pelvic position. Less frequently, the appendix may adopt other positions such as pre- or post-ileal (5%), subcecal, paracaecal (alongside the cecum), or near the sacral promontory [4,5,6,7]. These positional variations can influence the clinical presentation and diagnosis of appendicitis, as the location of pain may differ depending on the appendix’s position within the abdominal cavity [7].

The evolutionary history of the appendix is fascinating and complex. Initially deemed an imperceptible organ with no apparent function, the appendix was thought to be a remnant of a larger cecal structure that aided in the digestion of cellulose when our ancestors had a more herbivorous diet [8,9]. However, this view has shifted dramatically in recent years. Research suggests that the appendix has grown autonomously in several mammalian lineages, indicating that it may serve an important purpose [1,2,8,10]. It appears that the appendix has been part of mammalian anatomy for at least 80 million years, much longer than Darwin’s theory would suggest [1]. This longevity across evolutionary history implies that the appendix may have conferred a selective advantage.

Emerging theories propose that the appendix’s microbiome and its interaction with the gut microbiome are crucial for maintaining a balanced immune response. One of the leading theories today is that the appendix acts as a reservoir for commensal bacteria (probiotics), playing a crucial role in repopulating the gut microbiome (GM) after episodes of gastrointestinal (GI) disease [11,12]. This function is supported by the existence of a dense concentration of lymphoid tissue within the appendix, which suggests an immunological role. This “backup” system underscores the appendix’s significance in gut health, suggesting that while we can live without it, its presence may offer subtle yet important benefits [11,12]. This lymphatic tissue can encourage the growth of useful gut bacteria and play a part in the body’s immune response. The appendix’s association with the immune system is further supported by its role in the development of B and T lymphocytes and the production of IgA antibodies. These functions are particularly active during the first few years of life, indicating that the appendix may play a role in the development of the immune system in young individuals [2,13,14].

Despite these findings, the exact function of the appendix remains a topic of debate. Some scientists argue that if the appendix were truly vital, its removal would result in more significant health consequences [14,15,16]. However, the lack of observable health problems following an appendectomy does not necessarily mean the appendix is useless; it may indicate that other parts of the body can compensate for its absence [15,16]. The appendix is an integral part of the immune system with a significant role in maintaining gut health. Its evolutionary persistence suggests that it provides a beneficial function, possibly related to the GM and immune responses [2,9,10]. As research continues, our understanding of the appendix’s role in human physiology will likely become clearer, potentially leading to new insights into its contribution to our overall health and well-being.

## 2. Appendix Microbiome Composition in a Healthy Person

Studies investigating the microbial composition of the human appendix have revealed a rich and diverse ecosystem predominantly comprising four major phyla: *Firmicutes*, *Bacteroidetes*, *Actinobacteria*, and *Proteobacteria* [17,18]. This microbial consortium exhibits dynamic interactions, forming intricate networks that influence various aspects of host physiology, including nutrient metabolism, immune modulation, and pathogen resistance [12,19,20].

*Firmicutes* are often the most abundant phylum within the AM. They are known for their role in fermenting indigestible carbohydrates, producing short-chain fatty acids (SCFAs) such as butyrate, propionate, and acetate [21,22]. Bacterial genera of this phylum include *Ruminiococcus*, *Clostridium*, and *Lactobacillus* [23]. These SCFAs serve as a primary energy source for colonocytes and exhibit anti-inflammatory properties, which are vital for maintaining intestinal barrier integrity and preventing the invasion of pathogens [21,24]. Moreover, *Firmicutes* contribute to the regulation of the host’s immune system. They influence the balance of pro- and anti-inflammatory cytokines, which are key mediators in immune response [24]. This modulation is essential for maintaining gut homeostasis and can impact the development and progression of various infectious and inflammatory diseases [24]. The *Firmicutes*/*Bacteroidetes* ratio is also a significant marker of gut health, changing throughout different life stages and reflecting the dynamic nature of the GM [25,26]. *Firmicutes* and *Bacteroidetes* account for 90% of human bacterial species [27]. Alterations in this ratio have been associated with various health conditions, including obesity and metabolic syndrome [28]. *Firmicutes* interact with the immune system by impacting the proliferation, growth, and differentiation of epithelial and immune cells [21,25]. This interaction is crucial for the proper functioning of the gut’s immune defense mechanisms. Both diversity and functionality in *Firmicutes* are essential for optimal gut health. Their ability to produce butyrate supports both immune regulation and the proper functioning of the intestinal lining, highlighting their importance in the overall health of the host [24,25,26,28].

*Bacteroidetes* are another significant group within the AM. Bacterial genera of this phylum include *Bacteroides*, *Prevotella*, and *Porphyromonas*, in which *Bacteroides* alone account for around 30% of all gut bacteria [27]. Bacteroidetes are renowned for their ability to degrade complex polysaccharides and proteins, facilitating digestion and nutrient absorption [12]. Their enzymatic machinery is specialized for breaking down dietary fibers, which are otherwise indigestible by human enzymes, thus playing a pivotal role in the host’s energy harvest [29]. Beyond digestion, *Bacteroidetes* are integral to gut health and immunity. They engage in colonization resistance, a process where they outcompete pathogenic bacteria for adhesion sites and nutrients, and produce bacteriocins to inhibit the growth of potential pathogens [30]. This competitive exclusion is crucial for maintaining a balanced GM and preventing infections. Moreover, *Bacteroidetes* influence the immune system by modulating the production of anti-inflammatory molecules and interacting with the gut’s mucosal immune system [31]. They help maintain the integrity of the intestinal epithelium, which serves as the first line of defense against external pathogens [32]. The mode of action of *Bacteroidetes* includes the stimulation of immune responses through the production of signaling molecules that can affect immune cell differentiation and function. This interaction is essential for the maturation of a strong immune system capable of responding to a variety of challenges [33,34].

*Actinobacteria*, while less abundant in the AM compared to *Firmicutes* and *Bacteroidetes*, play a significant role in human health [35]. Bacterial genera of this phylum include both anaerobic and aerobic bacterial including *Bifidobacteria*, *Propionibacteria*, *Corynebacteria*, *Streptomyces*, *Nocardia*, *Micromonospora*, *Actinoplanes*, and *Streptosporangium*, of which *Bifidobacteria* are most common in the human gut [36]. They are adept at degrading complex sugars, which is crucial for the digestion of dietary fibers that human enzymes cannot process. This degradation process results in the production of bioactive compounds, including various vitamins and anti-inflammatory substances [37]. *Bifidobacteria*, a well-known genus within the *Actinobacteria* phylum, are widely recognized as probiotics. They contribute to gut health by enhancing the intestinal barrier, modulating the immune system, and producing lactic acid, which lowers the pH of the gut environment, inhibiting harmful bacteria [38]. Furthermore, *Bifidobacteria* can synthesize essential vitamins such as biotin, folate, and vitamin K, which are vital for the host’s metabolism [35,39]. The mode of action of *Actinobacteria* involves not only the breakdown of complex carbohydrates but also the interaction with the host’s immune cells. They can influence the maturation and function of immune cells, thereby playing a role in both innate and adaptive immunity. This interaction is particularly important for the development of immune tolerance and the prevention of inflammatory diseases [40]. Recent studies have also highlighted the role of *Actinobacteria* in aging, showing that their abundance and diversity can change with age, impacting the overall health of the individual [37].

Proteobacteria are a major group (phylum) of bacteria that includes different pathogens, for instance, Brucella, Rickettsia, Bordetella, Neisseria, Escherichia, Salmonella, Vibrio, and Helicobacter [32,41]. Proteobacteria form a minor portion [42]. Desulfovibrio, Escherichia, Klebsiella, and Shigella are the major representative Proteobacteria in the intestinal microbiota. They form a minor portion of the GM, and are most unstable [43]. While some members are opportunistic pathogens, many non-pathogenic Proteobacteria are beneficial, contributing to microbial diversity and performing essential metabolic functions [44]. These beneficial Proteobacteria are involved in nitrogen fixation, an important process for recycling nitrogen within the gut ecosystem [45]. They also participate in the breakdown of complex molecules, which can contribute to nutrient cycling and energy production within the gut [37,44]. Moreover, Proteobacteria can influence the immune system. They are involved in the production of molecules like lipopolysaccharides, which can modulate immune responses. Some Proteobacteria are known to stimulate the production of anti-inflammatory cytokines, helping to maintain a balanced immune response and protect against inflammatory diseases [46]. The mode of action of Proteobacteria in the gut includes their interaction with other microbes and the host’s immune cells. They can compete with pathogenic bacteria, preventing their overgrowth and colonization. This competitive interaction is crucial for maintaining a healthy GM and preventing infections [47].

Overall, the AM in a healthy person is a complex and dynamic community that plays a significant role in gut health and the immune system. The four major phyla, *Firmicutes*, *Bacteroidetes*, *Actinobacteria*, and *Proteobacteria*, contribute to the functionality of the GM, with implications for the gut–brain axis and overall well-being. Understanding the composition and function of the AM is vital for the development of strategies to maintain gut health and prevent diseases related to microbial imbalances.

## 3. Appendix Versus Gut Microbiome

The human appendix and gut are both integral components of the digestive system, each with distinct roles and characteristics. However, there are some differences between AM and GM that are discussed in more detail below (Table 1).

One of the key differences between the appendix and the GM is the formation of biofilms. In the appendix, biofilms are robust and play a crucial role in protecting and preserving the microbiome [55]. In the gut, biofilms also exist but are typically less dense and more susceptible to disruption. When the GM is depleted, the appendix can release bacteria from its biofilms to repopulate the gut, thus maintaining a healthy microbial balance [56].

## 4. Immunological Function of Appendix and Appendix Microbiome

### 4.1. Immunological Roles

**Figure 1:**
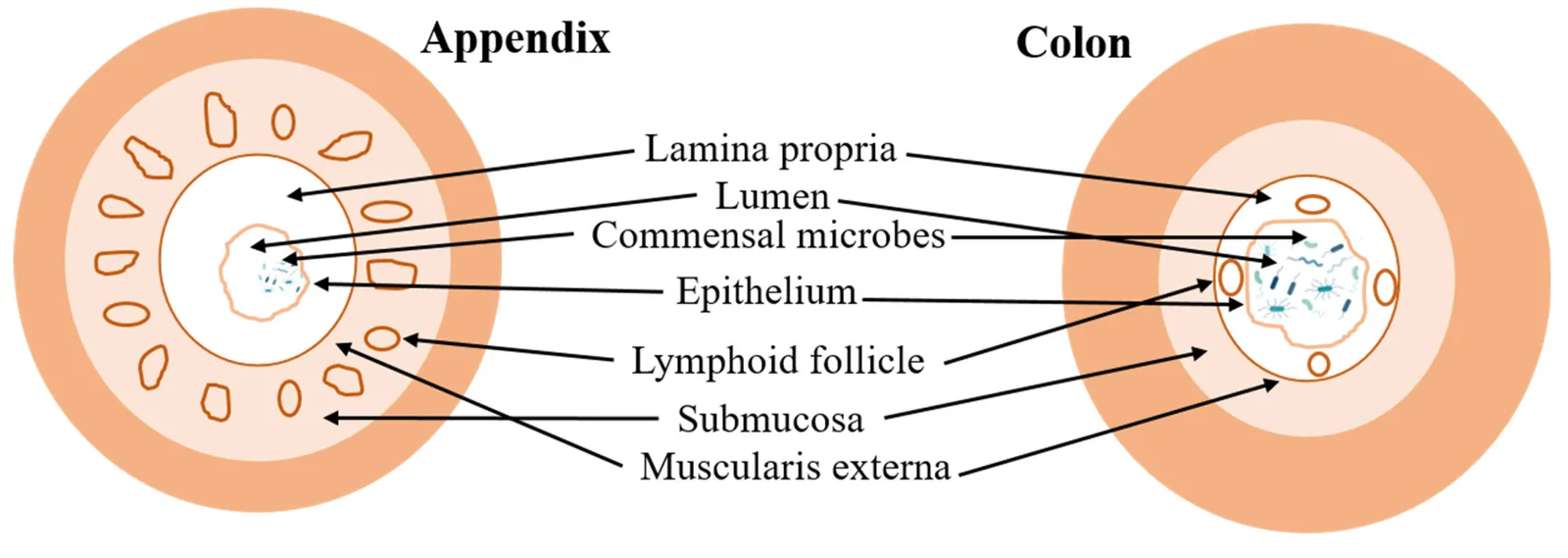
A schematic diagram representing the difference between the appendix and colon layers. The figure shows that the appendix has more abundant and pronounced lymphoid follicles and a different microbiome composition compared to the colon.

The appendix is rich in lymphoid tissue, which plays an immunological function. Lymphoid tissue plays a crucial role in promoting the growth of beneficial gut bacteria (i.e., *Blautia* sp., *Enterococcus hirae*, *Lachnospiraceae bacterium*, *Collinsella aerofaciens*) (Figure 1) [52]. The appendix provides a protected environment for these beneficial bacteria [52]. Within the appendix, lymphoid tissue forms a network of structures called follicles, which contain immune cells like B and T lymphocytes [57]. These immune cells are essential for the immune response but appear to be more tolerant towards beneficial bacteria in the appendix than in the main gut. This tolerance allows beneficial bacteria to reside and potentially replicate within the appendix, acting as a reservoir [52].

Furthermore, the appendix might play a role in “educating” the immune system to distinguish between beneficial and harmful bacteria [2]. By encountering diverse bacterial populations within the appendix, the immune system can learn to tolerate beneficial bacteria while remaining vigilant against pathogens [10]. Such immune training may help the body tolerate harmless antigens, and loss of this tolerance may lead to allergies and autoimmune diseases (ADs) [58,59].

### 4.2. Interaction with Immune System

The appendix, on the other hand, interacts with the host’s immune system mainly by gut-associated lymphoid tissue (GALT), a network of immune cells concentrated in the appendix and other intestinal tissues which comprise different immune cells like dendritic cells (DCs), macrophages, and innate lymphocytes that serves as the first line defense (Figure 1) [17,56,60]. Specific commensal bacteria like *Bifidobacteria* within the appendix can stimulate the development and function of GALT [61]. Moreover, *Bifidobacteria* are associated with oncogene suppression and immune modulation [62,63]. *B. longum* may suppress azoxymethane-induced colon tumors by decreasing colonic mucosal proliferation and ornithine decarboxylase and ras-p21 activity [63]. They may help decrease the activity of different pro-carcinogenic enzymes such as β-glucosidase and β-glucuronidase [62]. *B. bifidum* was reported to decrease the activity of β-glucosidase [62], and *B. longum* lowered the activity of β-glucosidase and β-glucuronidase [64]. This highlights the potential role of the AM in both gut homeostasis (stable internal environment) and disease pathogenesis (disease development) mediated by microbes and the immune system [13,65]. Studies using germ-free mice (mice raised in a sterile environment devoid of microbiota) demonstrate the critical role of gut bacteria in GALT development. *Bifidobacterium pseudolongum*, *Lactobacillus johnsonii*, and *Olsenella* significantly improved the efficiency of immune checkpoint inhibitors in four cancer mouse models. *B. pseudolongum* modified improved immunotherapy responses by way of inosine production [66]. This observation underscores the dependence of GALT on microbial stimulation for proper development and function in animals [56,67].

**Figure 2:**
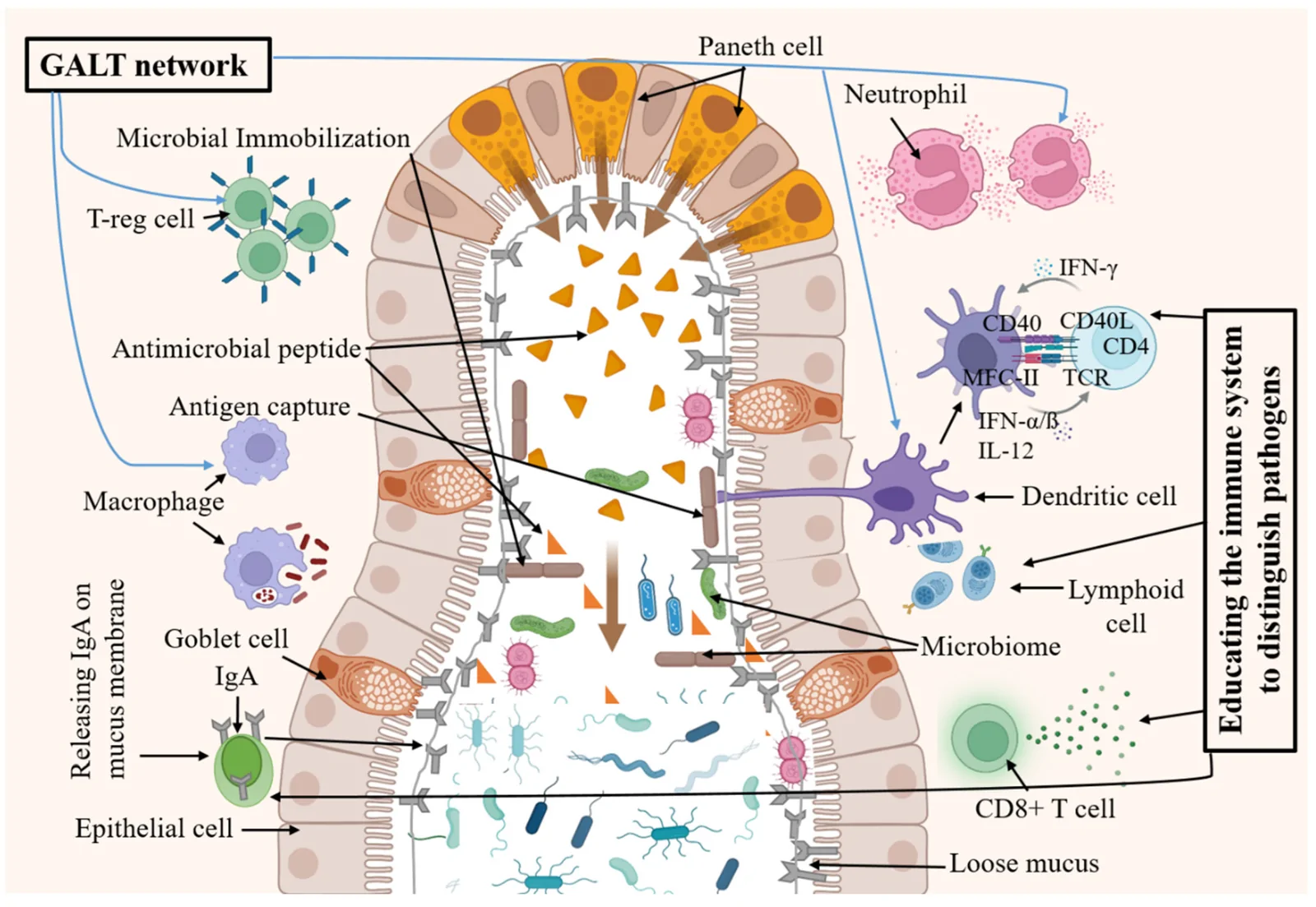
Immunological function of the appendix. Lymphoid tissue of the appendix forms a GALT (gut-associated lymphoid tissue) network using macrophages, neutrophils, dendritic cells, and T cells. Moreover, lymphoid cells and Treg cells help to educate the immune system to counter the pathogens upon infection.

There are several ways in which AM can interact with the immune system. Certain bacterial strains within the AM can promote the development of Tregs, a specialized subset of immune cells that suppress excessive immune responses and maintain tolerance to commensal bacteria. For instance, *Lactobacillus rhamnosus* stimulates the cell proliferation rate of bowel epithelial cells, enhances repairing of the mucosa damage caused by radiotherapy and/or chemotherapy, helps maintain bacterial equilibrium within the bowel, and inhibits bacterial translocation into the tissues [68,69,70]. This helps prevent autoimmune reactions against the body’s own tissues [71,72]. In response to *Lactobacillus*, epithelial cells increase the extracellular expression of MUC3 mucin which limits the adhesion of pathogens like enteropathogen *E coli* E2348/69 [69]. A similar result was reported with *Lactobacillus casei GG* which reduces the adhesion of *E. coli* C25 in epithelial cells [68]. The appendix’s lymphoid tissue provides a niche for the maturation of B and T lymphocytes, the key players in the adaptive immune response. These immune cells learn to recognize and respond to foreign antigens (substances that trigger an immune response) while remaining tolerant to self-antigens [73]. The appendix epithelium produces significant amounts of IgA, a type of antibody that neutralizes pathogens on mucosal surfaces (surfaces like the lining of the gut) and prevents them from adhering and causing infection (Figure 2) [74]. This localized immune response within the appendix complements the broader immune activities of the gut. Defects in IgA production cause increases in specific bacterial taxa like *Bacteroides* in the progression of colitis [75]. These findings are also supported by recent studies. In one recent study, the association of appendectomies with an increased risk of colitis-associated cancer (CAC) was examined using five-week-aged male BALB/c mice. The results showed that UC patients with a previous appendectomy had decreased intratumor CD3+ and CD8+ T-cell densities compared with UC patients without a previous appendectomy [76].

Studies suggest that an altered microbial composition may trigger an inappropriate immune response against commensal bacteria, leading to intestinal tissue damage [77]. Manipulating the AM can promote the development of regulatory T cells (Tregs) and suppress autoimmune reactions [78,79]. Additionally, appendix-associated microbes may aid in restoring the GM following antibiotic use or infections [17].

## 5. Appendectomy’s Link to Disease Outcome

**Figure 3:**
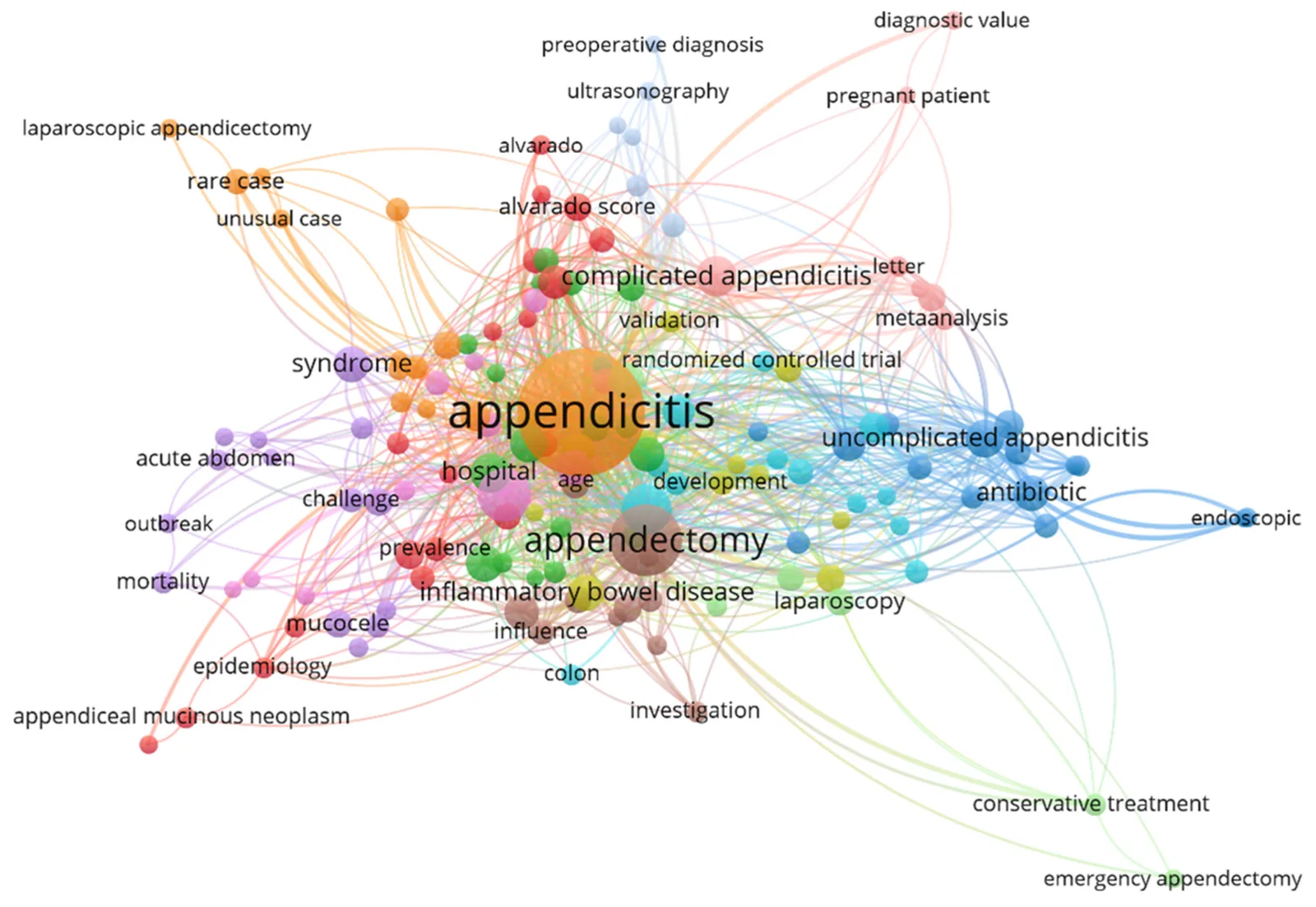
Bibliometric analysis of appendectomy literature and keyword analysis. Data on the impact of appendectomy on different disease outcomes were retrieved from the PubMed database. This VOSviewer network analysis, conducted using VOSviewer version 1.6.20 and the dimensions research publication database, considers 1720 published studies from the period 2020 to 2024 on appendectomy and related diseases. The analysis was performed based on 148 keywords selected from these publications, and 12 found distinct clusters were found within key terms. The visualization illustrates the interconnectedness of various terms in appendicitis research, highlighting key themes such as diagnostic tools, treatment approaches, and emerging trends. Central terms like appendicitis, appendectomy, and differentiations between complicated and uncomplicated appendicitis are featured. The network also underscores the geographical and temporal diversity of the research, emphasizing the collaborative efforts in addressing appendicitis. This comprehensive network analysis provides valuable insights into the evolving appendicitis research and its implications for advancing diagnostic and treatment strategies.

While appendectomies are generally considered safe, some studies suggest they may be associated with an increased risk of certain conditions. Patients who have undergone appendectomy might have a greater risk of developing inflammatory bowel diseases (IBDs) like Crohn’s disease (CD) [14]. Additionally, there is a noted higher risk of infections like *Clostridium difficile* and sepsis following the procedure [80,81]. Though the evidence is inconclusive, some research has also explored a potential link between appendectomy and an increased risk of CRC [82,83]. Figure 3 shows the VOSviewer 1.6.20 network analysis combining insights from related research publications on appendectomies and related disease outcomes. Moreover, changes in the composition of the AM have been linked to specific GI disorders, underscoring its significance in human health [17,19]. Advancements in sequencing technologies and bioinformatics analyses have modernized our understanding of the diversity and roles of appendix-associated microbes [17]. Metagenomics studies have unveiled their ability to metabolize diverse dietary compounds, synthesize vital vitamins, and modulate the host’s immune system, highlighting the multifaceted functions of the AM [84,85,86,87].

### 5.1. Gut Dysbiosis

The GM, a complex ecosystem of trillions of microorganisms including bacteria, viruses, fungi, and protozoa, primarily resides in the GI tract, with the highest concentration found in the large intestine [11]. Recent research has shifted perspectives on the human appendix, highlighting its significant role in regulating the intestinal immune system and maintaining a healthy gut microbiome [19]. This function is particularly important in the context of the gut–brain axis, where the GM has been shown to influence brain function and behavior, potentially impacting neurodevelopmental and psychiatric disorders [37,42,88,89].

**Figure 4:**
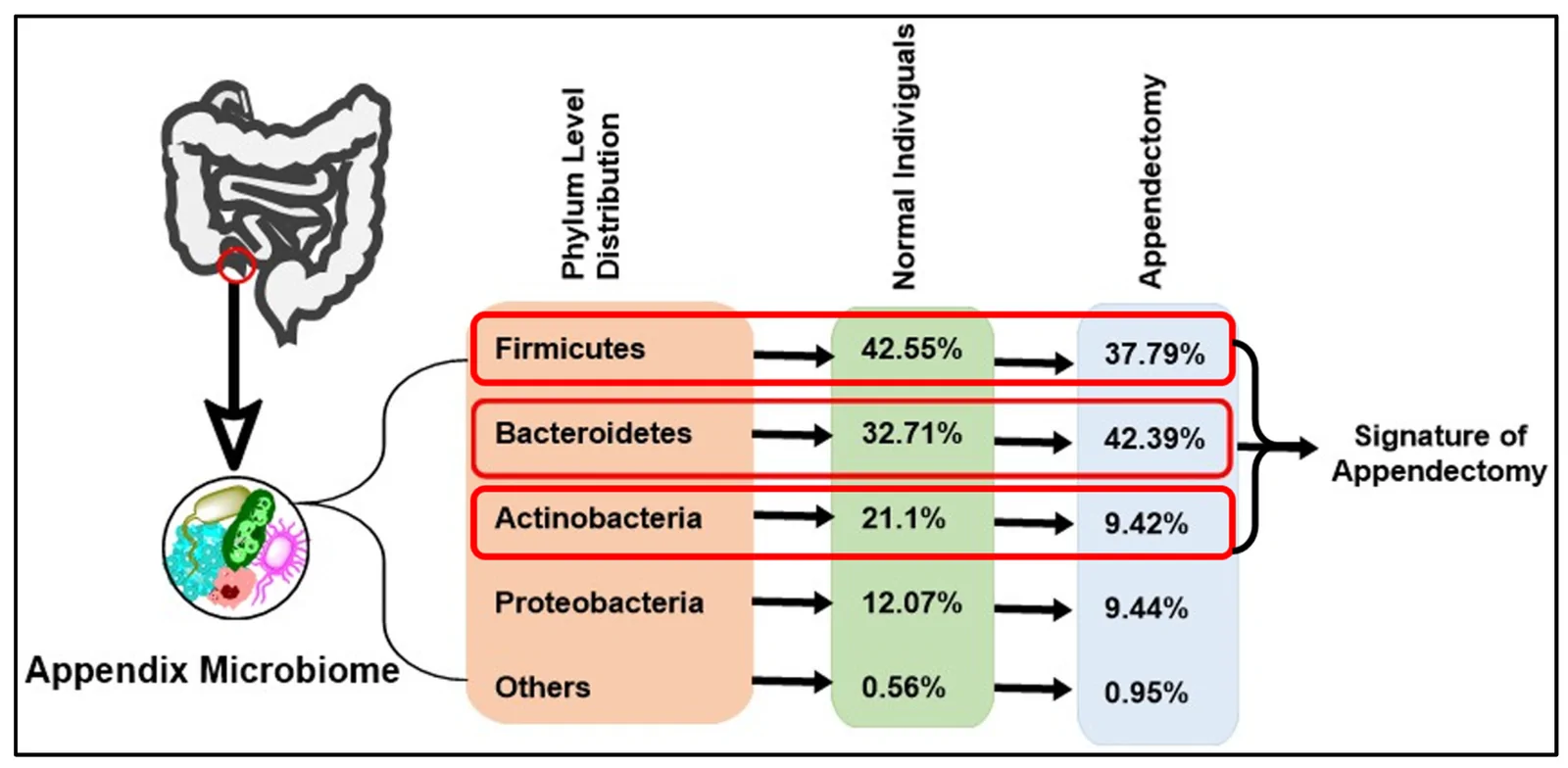
Compositional changes in major phyla of GM after appendectomy. The figure shows the major changes in phylum level before and after appendectomy. Highlighted the top 3. It shows that bacteria under phylum *Bacteroidetes* increase heavily, whereas other phyla show a decrease in their number and diversity. As a result, *Firmicutes*/*Bacteroidetes* change significantly after an appendectomy, which is the key cause of many dysbiosis-related disease progressions.

The impact of appendectomy (surgical removal of the appendix) on the GM has been a focus of recent studies. A cross-sectional study comparing individuals with and without a history of appendectomy (HwA and HwoA, respectively) revealed key differences in their gut microbial compositions; HwA individuals exhibited reduced microbial diversity, with lower abundance of beneficial SCFA-producing microbes such as *Roseburia*, *Barnesiella*, *Butyricicoccus*, *Odoribacter*, and *Butyricimonas* [19]. Specifically, beneficial microbes such as *Roseburia*, *Barnesiella*, *Butyricicoccus*, *Odoribacter*, and *Butyricimonas* were less abundant in HwA samples, which means the appendix has some special microbial content [19]. These microbes are known for producing SCFAs, which are crucial for gut health. This shift in microbial populations can have far-reaching effects on gut health, potentially increasing susceptibility to GI diseases and affecting the body’s immune response [19]. Although there is a tendency for the GM to gradually restore its diversity over time post-appendectomy, significant differences remain [19,52]. Notably, HwA individuals showed higher gut fungal diversity and more complex fungal–bacterial interactions even five years after appendectomy [52,90,91]. Moreover, the appendix is thought to have a higher concentration of certain beneficial bacteria, such as *Bacteroidetes* and *Firmicutes* [91,92,93] (Figure 4). These bacteria are essential for various functions, including the fermentation of indigestible fibers and the production of SCFAs, which are vital for colon health [2]. Beneficial bacteria, such as *Lactobacillus* and *Bifidobacterium*, are essential for maintaining a healthy GM. These “backup” probiotics are then released from the appendix to restore balance within the large intestine, aiding in recovery and maintaining gut health [19,65,91].

### 5.2. Risk of Colorectal Cancer (CRC)

Emerging research has established a link between appendectomy and an elevated risk of CRC, believed to be influenced by changes in the GM following the procedure [52,94,95]. After an appendectomy, there is a notable increase in CRC-promoting bacteria such as *Bacteroides vulgatus*, *Bacteroides fragilis*, *Veillonella dispar*, *Prevotella ruminicola*, *Prevotella fucsa*, *Prevotella dentalis*, and *Prevotella denticola*. These bacteria are associated with promoting colorectal carcinogenesis [52,96]. Conversely, beneficial commensals like *Blautia* sp. YL58, *Enterococcus hirae*, *L. bacterium* Choco86, *C. aerofaciens*, and *Blautia* sp. SC05B48 are found to be depleted in individuals who have undergone an appendectomy [52,96]. These commensals are generally associated with maintaining gut health, and their reduction can disrupt the GM balance.

This shift in the GM, marked by an increase in CRC-promoting bacteria and a decrease in beneficial commensals, suggests that GM may play a vital role in the development of CRC caused by appendectomy [95,97]. The appendix contains substantial lymphatic tissue associated with immune function, and some studies suggest it may have a protective effect against colorectal carcinoma due to its immunological role [94,98]. However, an appendectomy remains the most frequently performed emergency surgical procedure, and recent evidence highlights its potential link to increased CRC risk [52,94,95,99]. Researchers continue to explore the impact of appendectomy on GM, which is crucial for maintaining intestinal health [94,95,98,99,100,101,102,103].

Dysbiosis may also contribute to CRC risk by promoting carcinogenesis through various mechanisms. Direct DNA damage is one of them. Dysbiotic changes in the GM can indeed lead to DNA damage in colonic cells. This process occurs during DNA replication when unrepaired DNA damage in the template strand arrests replication fork progression, ultimately resulting in fork collapse, double-strand break formation, and genome instability [104,105,106,107,108]. Dysbiosis may cause inflammation, which later induces immune cells to release Reactive Oxygen Species (ROS) and Reactive Nitrogen Species (RNS), and cause DNA damage. Moreover, GM converts many metabolites from the diet into DNA-damaging agents or oncometabolites. These types of metabolites include p-cresol, secondary bile acids, or N-nitrosamines, which favor mutagenesis and CRC development [105]. Moreover, genotoxic metabolites produced by some GM promote CRC. For instance, colibactins, hybrid polyketide-nonribosomal peptides, synthesized by *E. coli*, *Klebsiella pneumoniae*, and other *Enterobacteriaceae* harboring the *pks* genomic island are genotoxic metabolites that have DNA damaging capability and promote CRC progression [107]. Altered GM may increase oxidative stress, which is linked to cancer development. While oxidative stress is a natural consequence of cellular metabolism, dysbiosis can exacerbate it, potentially contributing to carcinogenesis [104,109,110,111].

A recent study reviewed medical records of 455 patients diagnosed with colorectal adenocarcinoma (CRC) over a five-year period [94]. The results showed the following: (1) Right colon adenocarcinoma (CA): appendectomy has been identified as the second-highest risk factor for right colon adenocarcinoma. According to the study, the risk of developing right colon adenocarcinoma was found to be significantly higher for individuals who had undergone an appendectomy [94]. (2) Left CA: The same study reported that appendectomy increased the risk by 2.537 times. This suggests a strong association between the surgical removal of the appendix and the development of adenocarcinoma in the left colon [94]. With regard to (3) rectum adenocarcinoma, the following was found: The risk of rectum adenocarcinoma was increased by 3.232 times following an appendectomy [94]. This indicates that appendectomy is a notable risk factor for adenocarcinoma in the rectum as well [94].

Recent research has challenged the conventional view that appendectomy increases the risk of cancer, particularly CRC. A prospective population-based cohort study found that a history of appendectomy was linked to a lower risk of cancer, including GI and colon cancers [112,113]. However, not all studies agree on this association; some suggest no overall increase in CRC risk or its subtypes among those who underwent appendectomy (Table 2) [98,114,115]. Additionally, appendectomy has been linked to improved clinical outcomes in UC but may intensify the risk of advanced colorectal neoplasia [116].

The topic remains complex, with researchers actively exploring the underlying mechanisms and considering various individual factors. It is crucial to evaluate both sides of the argument and stay informed as new findings emerge. Although mechanisms are not yet clear, current evidence underscores the need for further investigation to clarify the underlying biology and challenge conventional views.

### 5.3. Risk of Inflammatory Bowel Diseases (IBDs)

Gut dysbiosis and alterations in microbial metabolic networks are closely associated with the pathogenesis of IBD [122]. Appendectomy has been associated with a reduced risk of UC, although its relationship with IBD remains complex, with an increased risk of CD reported in some epidemiological studies [65,100]. This complexity highlights the ongoing debate regarding whether it is the appendectomy procedure itself or underlying appendicitis that influences IBD susceptibility and progression. Several studies suggest that following an appendectomy, the prevalence of UC was lower, whereas the prevalence of CD was higher than in controls [98].

Regardless of appendicitis incidence, some studies reported that appendectomies might increase the risk of UC and CD [123,124,125,126]. Compared to the comparator cohort, the appendectomy cohort showed a 2.23- and 3.48-fold increased risk of UC and CD (adjusted HR = 3.48, 95% CI = 2.42–4.99), respectively [123]. Notably, the relationship between appendectomies and CD appears to be time-dependent, with an increased risk observed particularly within the initial years following surgery, after which the risk declines over time [127]. A similar trend has been reported in other studies [100,128]. For instance, an appendectomy being performed between the ages of 18 and 29 has been especially linked to a higher incidence of CD (HR = 2.02; 95% CI: 1.66 to 2.44), reinforcing the need to consider the age at surgery when evaluating CD risk [100]. Furthermore, long-term analyses show that the risk of developing CD remains elevated even five years post-appendectomy (RR = 1.24, 95% CI: 1.12–1.36) [128]. Although these studies show a higher risk of CD after appendectomy, it remains unclear whether the surgery itself is the cause. An early international case–control study found that appendectomy was more common in patients with CD than in controls, but the study suggested that some of these operations may have been related to CD itself [129]. Some studies suggest that patients who underwent appendectomy due to appendicitis had a modestly increased risk of CD (RR = 1.64, 95% CI: 1.17–2.31), whereas those who had an appendectomy without appendicitis exhibited an even higher risk (RR = 2.77, 95% CI: 1.84–4.16) [125]. This distinction is crucial, as it suggests that the inflammatory process of appendicitis itself, rather than the surgical removal of the appendix, may contribute to disease development.

Scientific evidence supports the potential role of the appendix in UC pathogenesis and suggests that its removal might influence disease progression [65,100]. In contrast to age at appendectomy, the risk of UC appears to decline with increasing time after surgery (HR = 0.21; 95% CI: 0.06 to 0.72, comparing ≥5 with 0–4 years after appendectomy) [100]. A similar trend has been noted in other studies. Importantly, appendectomy has been found to exert a protective effect against UC, with some reports indicating that the more time elapses post-surgery, the stronger the protective effect [100]. Moreover, in patients already diagnosed with UC, appendectomy has been associated with a reduction in disease severity, suggesting a potential therapeutic benefit in certain cases [130]. This interplay between the appendix, gut microbiota (GM), and the immune system further complicates our understanding of IBD risk. Emerging research suggests that appendectomy may influence immune modulation and microbial composition, which could subsequently alter IBD pathophysiology. Furthermore, studies investigating the risk of advanced colorectal neoplasia in IBD patients who have undergone appendectomy provide valuable insights into the long-term consequences of this surgical intervention [116]. The impact of appendectomy on IBDs is multifaceted and varies depending on the type of IBD, patient age at surgery, underlying appendicitis status, and time elapsed post-appendectomy (Table 3). While appendectomy appears to be protective against UC, its relationship with CD is more complex and requires further investigation to clarify whether it is appendectomy itself or the inflammatory process of appendicitis that influences CD risk.

### 5.4. Recurrent Clostridium difficile Associated Colitis

An appendectomy may increase susceptibility to *Clostridium difficile* (*C. difficile*) colitis due to changes in the GM [138]. *C. difficile* colitis occurs due to the overgrowth of *C. difficile* bacteria, typically following the disruption of normal gut flora by antibiotic use [139]. Recurrent *C. difficile* colitis is a significant issue in modern medicine, with hospital-acquired cases leading to increased mortality rates [140,141]. While it might be expected that the appendix would protect against *C. difficile* overgrowth, it is uncertain if the appendix can shield beneficial bacteria from the broad-spectrum antibiotics that precede *C. difficile* colitis. The appendix may have evolved to help recover from infections, but its protective role against modern antibiotics remains doubtful [80].

However, strong evidence from a recent study indicates that the appendix may indeed play a protective role in recurrent *C. difficile* colitis [81]. A previous study found that patients without an appendix had a 2.5-fold increased risk of recurrent *C. difficile* colitis compared to those with an appendix. This suggests that the appendix may protect normal gut flora from treatments like oral vancomycin, used for recurrent *C. difficile* colitis [80]. Clinical data indicate that while appendectomy increases the risk of recurrent *C. difficile* colitis and potentially other microbiome-related diseases, it does not increase the risk of the initial onset of *C. difficile* colitis (Table 4) [80]. These observations suggest that the appendix might not effectively protect the microbiome from broad-spectrum antibiotics that lead to the initial onset of *C. difficile* colitis.

### 5.5. Risk of Parkinson’s Disease (PD)

The appendix has been implicated in the gut–brain axis, potentially influencing the pathogenesis of PD through its effect on GM and the immune system. There is conflicting evidence on the impact of GM on PD. For instance, research has suggested that appendectomy might reduce the risk of developing PD [147]. A substantial study involving health records of around 1.7 million individuals found a 19.3% reduced cumulative incidence of PD among those who had undergone appendectomy [148]. This could be attributed to the removal of the appendix, which harbors alpha-synuclein aggregates, a pathological hallmark of PD [149]. The presence of these aggregates in the appendix, even in individuals without PD, implicates the organ as a potential source of neurodegenerative triggers [147,150]. Conversely, other studies have reported an increased risk of PD following an appendectomy. Analysis of health data revealed that individuals who had undergone an appendectomy were over three times more likely to develop PD later in life [151]. This suggests that the appendix might have a protective role against PD, possibly through the regulation of GM and immune surveillance [152]. Some studies reported that appendectomy may have no relation with PD. The debate over the role of appendectomy in PD is fueled by studies with conflicting outcomes. Some studies, like those conducted by Yilmaz et al. (2017) and Marras et al. (2016), did not find a significant correlation between appendectomy and the onset or clinical progression of PD [153,154]. Yilmaz et al. (2017) retrospectively analyzed 1625 patients and concluded that appendectomy did not influence the emergence or clinical characteristics of PD [153]. Similarly, Marras et al. (2016) investigated a large cohort of nearly 43,000 individuals and also reported no association between appendectomies and decreased risk of developing PD [154]. These findings suggest that the appendix’s removal does not necessarily alter the disease’s trajectory (Table 5).

### 5.6. Risk of Amyotrophic Lateral Sclerosis (ALS)

The relationship between appendectomies and the risk of developing ALS has garnered significant interest in recent research [163,164]. Although studies in this area are still ongoing, several important observations have been made. Some research suggests a potential protective effect of appendectomies, indicating a reduced risk of ALS, while other studies find no significant association between the two [164]. The mechanisms behind any potential link are not yet clear, but it is hypothesized that GM, which plays a crucial role in immune regulation, could be a contributing factor. ALS primarily affects individuals aged 55 and older, although it can occur at any age. While the connection between appendectomy and ALS risk is not fully understood, ongoing research aims to uncover potential links.

### 5.7. Risk of Auto-Immune Disease (AD)

Emerging epidemiological research found a potential link between appendectomies and the onset of AD. A pivotal study published in the Journal of the American Medical Directors Association (JAMDA) studies a comprehensive database from the Taiwan National Health Insurance Research Database to track individuals aged 45 and above who experienced acute appendicitis or underwent appendectomy [159]. Spanning an impressive follow-up duration of over 15 years, this research uncovered a heightened occurrence of AD in patients with a history of appendicitis or appendectomy compared to a matched control group [159]. Notably, the onset of dementia in these patients was significantly earlier, with a mean age of diagnosis at 70.18 years, roughly 5.84 years post-appendicitis event [159]. These observations hint at a potential association between appendectomies and an elevated risk of AD.

Further bolstering this association are studies examining the gut–brain axis, which suggest that modifications in the GM may play a role in AD pathogenesis [159]. Clinical evidence indicates that dysbiosis could influence brain health, potentially contributing to AD development [165]. In animal models, the transfer of healthy microbiota has been shown to mitigate amyloid and tau pathology, which are hallmark features of AD, suggesting that restoring gut microbial balance could be a therapeutic strategy [166]. Moreover, soluble alpha-synuclein, a protein implicated in neurodegenerative disorders, has been identified as a novel modulator of AD pathophysiology. Elevated levels of soluble alpha-synuclein may influence cognitive function and are associated with the aggregation of AD-related proteins [101].

Beyond AD, the appendix has also been implicated in other autoimmune and chronic inflammatory conditions. The appendix plays a key role in immune regulation, acting as a reservoir for commensal gut microbiota and influencing systemic immune responses [117]. Studies suggest that its removal may contribute to immune dysregulation, potentially increasing susceptibility to conditions such as rheumatoid arthritis, Crohn’s disease, and systemic lupus erythematosus (SLE) [167,168]. Moreover, its role in bacterial infections, particularly *Mycobacterium tuberculosis*, has been noted, with research indicating that appendiceal tuberculosis, though rare, may be an underrecognized manifestation of extrapulmonary tuberculosis [169].

The interplay between gut health, immune response, and neurodegeneration underscores the complexity of AD and highlights the need for comprehensive research. Emerging evidence suggests a possible link between appendectomy and an increased risk of AD, but these results should be approached with caution. The mechanisms underlying this association remain unclear, necessitating further investigation to determine causality and elucidate the biological pathways involved. The convergence of epidemiological data and preclinical insights offers a promising avenue for understanding the etiology of AD and exploring novel preventive and therapeutic approaches.

### 5.8. Risk of Heart Disease

According to earlier research, genetically anticipated appendectomies may increase the chance of developing ischemic heart disease (IHD) and its varieties, angina pectoris (AP) and acute myocardial infarction (AMI) [170,171,172]. Using Mendelian randomization (MR) research techniques and meta-analysis, the study demonstrated a strong positive causal link between appendectomy and IHD and its varieties, AMI and AP. IHD (OR: 1.128, 95% CI: 1.067–1.193, *p* = 2.459 × 10^−5^), AMI (OR: 1.195, 95% CI: 1.095–1.305, *p* = 6.898 × 10^−5^), and AP (OR: 1.087, 95% CI: 1.016–1.164, *p* = 1.598 × 10^−2^) were all positively correlated with appendectomies, according to the paper [170]. An elevated risk of recurrent IHD within three years following appendectomy was observed by another researcher [171]. They revealed that having an appendectomy at the age of 18 or older was independently linked to a 1.54-fold higher risk of IHD throughout the course of the 3-year follow-up (95% CI = 1.29–1.84) [171].

Childhood appendectomy and risk for premature acute myocardial infarction (AMI) was accessed in a cohort study. With adjusted HRs of 1.33 [95% CI: 1.05–1.70] for appendectomy, surgeries performed before the age of 20 were linked to a higher risk of AMI (417 occurrences as a result of the appendectomy). Additionally, the study indicated that there was no correlation between the incidence of AMI with an appendectomy performed at or after the age of 20 [173]. According to a recent study, children with asthma are more likely to have appendicectomies than people of all ages. Compared to the control group, the appendectomy group had increased adjusted risks of asthma (adjusted OR 1.18, 95% CI 1.13–1.23, *p* < 0.001) [174].

### 5.9. Risk of Type 2 Diabetes

Appendectomy increases type 2 diabetes risk, but as an age-dependent consequence [172]. A cohort study reported that individuals who had undergone appendectomy had a 7.9% greater overall incidence of type 2 diabetes than individuals who did not have the procedure. Compared to individuals who did not have an appendectomy, those under 30 years of age had an adjusted hazard ratio (HR) of 1.347 for type 2 diabetes (95% CI, 1.009–1.798). Compared to individuals who did not have an appendectomy, the incidence of type 2 diabetes was greater within three years of the follow-up (HR, 2.017; 95% CI, 1.07–3.802). In contrast, sex had no effect on the link between appendectomy and type 2 diabetes risk (*p* < 0.88), while age had a significant influence (*p* < 0.002) [175].

Patients in the appendectomy group who had preoperative blood glucose (POBG) values of ≥123 mg/dL (adjusted relative risk [aRR] 1.19; 95% CI 1.06–1.33) were 19% more likely than those with POBG levels of <106 mg/dL to have a hospital length of stay (LOS) of more than three days. The study showed that among patients having an appendectomy, a higher POBG level was significantly linked to a longer LOS [176].

### 5.10. Risk of Other Complications

Laparoscopic appendectomy (LA) is a new surgical procedure used to remove an infected appendix. Following a laparoscopic appendectomy, surgical site infections (SSI) are prevalent and can lengthen hospital stays. According to recent research, 45 patients (10.9%) experienced incisional SSI overall following LA. The probability of incisional SSI was independently correlated with the visceral fat area (HR 1.015, 95% CI 1.010–1.020, *p* < 0.001) [177].

According to certain research, the appendectomy groups had a higher pregnancy rate (first birth or first pregnancy) than the control groups [178,179,180]. For the appendectomy groups, the adjusted HRs for first pregnancy events that showed higher pregnancy rates were 1.20 (95% CI: 1.10–1.31) [179], 1.34 (95% CI: 1.32–1.35) [180], and 1.54 (95% CI: 1.52–1.56) [178]. Another study found that the pregnancy rate was higher following appendectomy for non-perforated appendicitis (HR = 1.11, 95% CI: 1.07–1.15) and after removing a normal appendix (HR = 1.48, 95% CI: 1.42–1.54), but not after perforated appendicitis (HR = 0.95, 95% CI: 0.88–1.04). In a recent study, open appendectomy was found to be safe for patients, but increased rates of cesarean delivery [181].

Studies have previously been conducted to assess the short-term and long-term mortality rates after appendectomy undertaking. The median overall fatality rate in the studies comparing open and laparoscopic procedures was 1.7% (range: 0.6–7.6%). Using predicted survival estimates from the Swedish population, the five-year follow-up study computed standardized mortality ratios [182]. The exposed cohort (appendectomy) saw fewer fatalities than anticipated when compared to the background population. With a standardized mortality ratio of 0.71 (95% CI: 0.67–0.75), the difference in mortality was particularly noticeable for non-perforated appendicitis. The median fatality rates for the open and laparoscopic procedures were 1.8% (range: 0.6–8.6%) and 0.9% (range: 0.3–3.6%), respectively. In two of the investigations, the laparoscopic group’s mortality rate was considerably lower than that of the control group [182,183]. Table 6 summarizes the research on appendectomy on heart disease, type 2 diabetes, and other complications.

## 6. Microbe-Based Therapies

Appendectomy leads to gut dysbiosis, resulting in IBDs, neurological disorders, ADs, and so on. Different methods aiming to adjust the gut microbial composition and restore dysbiosis have been explored as a means of managing the clinical conditions caused by gut dysbiosis. Examples of such methods include probiotics, prebiotics, postbiotics, symbiotics, fecal microbiota transplantation (FMT), phage-based therapeutics, and engineered microbes [195].

Probiotics are good bacteria and yeast that are beneficial to the health. Prebiotics are high-fiber foods that help the growth of beneficial intestinal microbes. Postbiotics are beneficial metabolites or byproducts produced by gut microbes that have various beneficial health impacts [195]. The use of prebiotics, probiotics, and postbiotics is of great interest to the scientific community for the treatment of complications associated with gut dysbiosis (Table 7). Extensive antibiotic use can disrupt the gut microbiota and contribute to the emergence of antibiotic-resistant pathogens [196]. The use of antibiotics during pregnancy may also impact the GM of infants, resulting in different GM-associated complications. A recent study showed that prebiotics in mothers could be an amazing alternative for reducing the disease severity in infants [197]. The use of multi-strain probiotics is one of the most-used microbe-based methods in clinical trials, which has a great impact on the functional diversity of the gut microbiota during pregnancy, reducing obesity and gastrointestinal complications in CRC patients, overcoming skin inflammation, and improving neurological disorders [197,198,199,200,201] (Table 7).

FMT has garnered a lot of interest and investigation lately as a dysbiosis remedy, and different disease management strategies. FMT directly affects the gut microbiota by transferring the complete intestinal microbiota from a healthy donor to the recipient in the form of feces using colonoscopy, enema, nasogastric (NG) tube, or capsule form [202,203,204]. Recent clinical trials showed that *L. acidophilus* transplanted by FMT reduced the cholesterol level in patients by inhibiting hepatic Cholesterol 7α-hydroxylase, restoring ileum Fibroblast growth factor 15 and small heterodimer partner proteins [205]. FMT of the mixed microbial population helped to reduce insulin resistance and interrupted CDI recurrence in the recipients [202,203].

**Table 7:** Microbe-based therapies under clinical trial to treat diseases caused by gut dysbiosis.

Therapy Method	Used Microbes	Disease Focus	Key Findings	References
Multi-strain probiotics	*Streptococcus thermophilus* NCIMB 30438, *B. breve* NCIMB 30441, *B. lactis* NCIMB 30435, *B. lactis* NCIMB 30436, *L. acidophilus* NCIMB 30442, *L. plantarum* NCIMB 30437, *L. paracasei* NCIMB 30439, and *L. helveticus* NCIMB 30440	Obesity	Pregnant women treated with multi-strain probiotics gave birth to a child who had decreased obesity-associated genera, *Collinsella*. Vaginally delivered infants had an increased number of *Bifidobacterium* and *Bacteroides*, and a decreased number of *Enterococcus* compared to the control group.	[197]
Multi-strain probiotics	*L. rhamnosus* (CECT 30031) and the cyanobacterium *Arthrospira platensis* (BEA_IDA_0074B)	Skin inflammation	Of the patients using the Global Acne Grading System, 17/40 (42.50%) were in the probiotic group (*p* = 0.02), indicating that the probiotic used in this study was effective and well accepted.	[199]
FMT	*L. acidophilus*	Cholesterol level	Inhibited hepatic Cholesterol 7α-hydroxylase, restored ileum Fibroblast growth factor 15, and small heterodimer partner.	[205]
Probiotics		Liver function	*L. acidophilus* supplementation promoted the recovery of liver function.	
Multi-strain probiotics	*B. longum*, *L. delbrueckii bulgaricus*, and *S. thermophilus*	Impact of probiotics on the functional diversity of the gut microbiota during pregnancy	Significantly increased the genera *Blautia*, *Ruminococcus*, and *Subdoligranulum* (*p* < 0.05). The functional genes of the gut microbiota involved in ABC transporters, oxidative phosphorylation, folate biosynthesis, and biotin metabolism were significantly increased in subjects receiving the probiotics (*p* < 0.05).	[198]
Probiotics	*B. longum* CECT 7347 (ES1)	Irritable bowel syndrome	Responder rates, stool consistency, abdominal pain severity, and anxiety, when compared to placebo over an 84 d time period.	[206]
Postbiotics	Heat-treated *B. longum* CECT 7347 (HT-ES1)			
Probiotics	*B. longum* NCC3001	Irritable bowel syndrome (IBS)	Improvements in anxiety and depression scores and a decrease in amygdala activation were observed. Moreover, the levels of butyric acid, tryptophan, N-acetyl tryptophan, glycine-conjugated bile acids, and free fatty acids were increased.	[201]
Probiotics	*B. breve M-16 V*	Human mode	*B. breve* M-16 V improved mood and sleep scores, decreased the heart rate under stress, and increased levels of pipecolic acid in stool samples, and improved mood and sleep scores in participants with high anxiety levels.	[207]
Probiotics	*Lacticaseibacillus rhamnosus* LRa05	*Helicobacter pylori* eradication	Did not improve *H. pylori* eradication significantly, but improved liver activity and reduced the levels of inflammatory markers like IL-6 and TNF-α.	[208]
Multi-strain probiotics	*B. infants*, *L. acidophilus*, *E. faecalis*, and *Bacillus cereus*	Gastrointestinal complications in CRC patients	Increased levels of *Bifidobacterium*, *Streptococcus*, and *Blautia*. Significantly Increased levels of SCFAs, mainly increasing acetate, butyrate, and propionate (*p* < 0.0001).	[200]
FMT	Mixed microbial population	Obesity	Significantly altered recipients’ phage and general microbial composition, suggesting that phages play an important role in changing the gut environment and thereby obesity.	[209]
Capsulized FMT	Mixed microbial population	UC	Remission induced in patients with UC by increasing the levels of *Alistipes* sp. and *Odoribacter splanchnicus*, and due to increased levels of indolelactic acid. Subjects without remission exhibited increased levels of *E. coli* and *Klebsiella*, and higher levels of 12,13-dihydroxy-9Z-octadecenoic acid and lipopolysaccharides.	[204]
FMT	Mixed microbial population	Insulin resistance	FMT with or without metformin significantly improved insulin resistance, body mass index, and gut microbial compositions of type 2 diabetes patients	[203]
FMT	Mixed microbial population	CDI	Recurrent CDI patients with sustained resolution after FMT had increased levels of *Ruminococcaceae* and *Lachnospiraceae*, and depletion of *Enterobacteriaceae*	[202]
Engineered bacteria	*E. coli* Nissle 1917 (EcN)	CRC	Oral administration of EcN as a probiotic increased their presence in the tumor cell the level of salicylate in the urine of adenoma-bearing mice was increased indicating that EcN could be used to treat CRC.	[210]

There have been conflicting outcomes where probiotics, prebiotics, postbiotics, and FMT are used. For example, some probiotic strains have been shown to help manage UC in certain randomized controlled studies, while CD has not consistently shown the same advantages [195]. It is difficult to draw firm conclusions on the efficacy of probiotics due to the wide variation in study designs, probiotic strains, doses, and patient demographics. Additionally, several studies have shown that FMT is useful in treating IBDs; however, the degree of success varies greatly. Furthermore, there is a continuous discussion about the long-term consequences and safety of microbiome manipulation, particularly when using aggressive techniques like FMT [211]. The possibility of spreading harmful bacteria as well as the long-term effects on the recipient’s immune system and metabolic functions are causes for concern.

## 7. Conclusions

Most of the research conducted on the impact of appendectomies on different disease outcomes involves statistical analysis, and there are very few experimental data on this topic. Moreover, much of the data found in this statistical research contradicts the experimental data found on the same topic. That is why more experimental data on the impact of appendectomies on different disease outcomes should be conducted. There are very few studies on the impact of appendectomies on neurological disorders (NDs) like depression, schizophrenia, PD, Amyotrophic lateral sclerosis (ALS), autism spectrum disorder (ASD), epilepsy, and migraines. More clinical and research data on these areas are desperately needed. Appendectomies are associated with some disease outcomes, but with different severities. Appendectomies are also linked to IBDs, but their impact on CD differs from UC. That is why it can be concluded that the association of appendectomies with different disease consequences is not uniform. Most of the disease consequences of appendectomies are caused by gut dysbiosis.

## Figures and Tables

**Table 1:** Differences between appendix and gut microbiome.

Feature	Appendix Microbiome	Gut Microbiome
Location	Narrow, finger-shaped pouch projecting from the cecum [48]	Extensive throughout the small and large intestines [49]
Diversity	Lower overall diversity compared to GM [17]	Higher overall diversity with a wider range of bacterial phyla [50]
Microbial Composition	Higher abundance of specific beneficial bacteria (*Firmicutes*, *Bacteroidetes*) [17]	More diverse range of bacterial groups (*Firmicutes*, *Bacteroidetes*, *Proteobacteria*, *Actinobacteria*) [51]
Function	Potential reservoir for beneficial bacteria, and immune system support [52]	Nutrient metabolism, digestion, immune function, and barrier function [50]
Biofilms	Forms robust biofilms that protect beneficial bacteria [2]	Biofilms present, but may be less dense and more susceptible to disruption [53]
Immune System	Rich in lymphoid tissue, supporting gut immune function [2]	Contributes to overall gut immune function [50]
Impact of Disruption	May contribute to diseases like appendicitis and IBD [19]	Can lead to various digestive issues, and metabolic disorders, and potentially influence overall health [54]
Impact on Disease	Potential link to appendicitis, IBD, CRC (research ongoing) [11,19]	Influences various GI diseases, metabolic disorders, and potentially neurological conditions [54]
Research	Emerging field of research, limitations exist	Extensive research area, well-established body of knowledge

**Table 2:** Recent research on the impacts of appendectomy on Colorectal Cancer (CRC) and other related cancers.

Disease Focus	Study Methodology	Key Findings	References
CRC	Review of existing research	Investigates the potential link between appendectomy and increased risk of CRC.	[117]
CRC	Analysis of appendix and GM in CRC patients and healthy controls	Dysbiosis was observed in both the appendix and GM of cancer patients, with specific bacterial signatures being identified.	[47]
CRC	Cohort study of 129,155 people	CRC risk increased 73% among appendectomy cases (Adjusted sub-distribution hazard ratio (SHR) 1.73, 95% CI 1.49–2.01, *p* < 0.001).	[52]
	Shotgun metagenomic sequencing technique	Significantly augmented 7 CRC-promoting bacteria (*B. vulgatus*, *B. fragilis*, *Veillonella dispar*, *P. ruminicola*, *P. fucsa*, *P. dentalis*, *P. denticola*) and the depletion of 5 beneficial commensals (*Blautia* sp. YL58, *E. hirae*, *L. bacterium* Choco86, *C. aerofaciens*, *Blautia* sp. SC05B48).	
CRC	Prospective cohort study of 139,406 participants	Appendectomy was linked to lower *F. nucleatum*-positive cancer occurrence (HR, 0.53; 95% CI, 0.33–0.85; *p* = 0.0079), but not *F. nucleatum*-negative cancer occurrence (HR, 0.98; 95% CI, 0.83–1.14).	[112]
CRC, colon cancer (CC), and rectum cancer (RC)	Mendelian randomization (MR)	Appendectomy did not increase risk of CRC, CC, or RC	[118]
CRC, colon cancer (CC), and rectum cancer (RC)	Prospective cohort studies and meta-analysis	Appendectomy was not linked to a higher risk of CC and RC, but a higher risk of CRC within a short time after surgery (1.68 [1.01–2.81]) was observed. The long-term risk was somewhat opposite (HR [95% CI], 0.94 [0.90–0.97]).	[119]
CRC	Multicentral CRC cohort	Previous appendix surgery is a sovereign risk factor for the diagnosis of patients with CRC through reductions in M1 macrophage cells.	[120]
CRC	Cohort of 75,979 patients	CRC frequency was 14% higher in appendectomy patients than in the contrast cohort (*p* < 0.05). Men were at higher risk than women. Patients ≥60 years old had an HR of 12.8 percent higher compared to those <60 years old.	[95]
Colitis-associated cancer (CAC)	Research on five-week-old male BALB/c mice after appendectomy	Intratumor CD3+ and CD8+ T-cell densities of UC patients were lower after appendectomy compared to the control.	[76]
Advanced colorectal neoplasia (aCRN)	A nationwide population-based cohort study	573 patients (1.4%) with appendectomy developed aCRN, with an absolute risk at 20 years of 4.9% (95% [CI], 2.9–7.7%) for ulcerative colitis (UC) patients. Appendectomies in UC patients were linked to increased rates of aCRN at 5–10 years (aHR, 2.5; 95% CI, 1.1–5.5) and 10–20 years (aHR, 2.3; 95% CI, 1.0–5.5).	[116]
Pancreatic cancer	Gene expression immune profiling, flow cytometry analysis, and microbiome sequencing	AM of pancreatic ductal adenocarcinoma (PDAC) patients showed abundances of *Klebsiella pneumoniae*, *Bifidobacterium animalis*, and *Adlercreutzia equolifaciens*.	[121]

**Table 3:** Recent research on the impacts of appendectomy on gut dysbiosis and IBDs.

Disease Focus	Methodology	Key Findings	References
Gut dysbiosis	Microbiome analysis of appendix tissue from appendectomy patients and healthy controls	Decreased bacterial diversity and enrichment of potential pathogens in appendicitis compared to controls.	[131]
Gut dysbiosis	16S rRNA gene sequencing of appendix tissue samples from appendectomy patients	Decreased bacterial diversity, enrichment of potential pathogens (e.g., *Escherichia coli*)	[12]
Gut dysbiosis	Using 16S and ITS2 sequencing of fecal samples	Subject without appendectomy had higher diversity with plenty of *Roseburia*, *Barnesiella*, *Butyricicoccus*, *Odoribacter*, and *Butyricimonas*.	[19]
IBD	Longitudinal analysis of appendix and GM in IBD patients and healthy controls	Distinct microbial composition in appendix of IBD patients compared to controls, suggesting potential role in disease pathogenesis.	[11]
IBD	Metagenomic analysis of appendix and gut tissue samples from IBD patients and healthy controls	Altered composition of AM compared to healthy controls, potential role in immune response	[132]
IBDs including CD and UC	Systematic review and meta-analysis	The incidence of UC and CD were 2.65 per 100,000 (95% CI: 1.39–3.90), and 1.16 per 100,000 (95% CI: 0.73–1.59). CD patients had a higher history of appendectomy Compared to UC patients.	[133]
CD and UC	Meta-analysis	The study revealed a significant association between appendicectomy and CD (OR: 1.57; 95% (CI): 1.01–2.43; heterogeneity I^2^ = 93%) and no significant association between appendicectomy and UC (OR: 0.60; 95% CI: 0.24–1.47; I^2^ = 96%),	[134]
CD	Cohort study of 21189 CD patients	Incidence of CD is milder for those who have previously undergone appendectomy.	[135]
CD	Statistical analysis of 2493 patients	Familial CD was linked to a higher rate of previous appendectomy history (*p* = 0.009) in East China.	[136]
UC	Nationwide prospective database study on 826 UC patients	Appendectomy is linked to 84% decreased risk of colectomy in patients having UC.	[130]
Colectomy and CRC in UC patients	Systematic review and meta-analysis on 73323 UC patients	Appendectomy in established UC is associated with apparently higher rates of subsequent CRC or high-grade dysplasia	[137]

**Table 4:** Recent research on the impacts of appendectomy on *C. difficile* associated colitis.

Disease Focus	Methodology	Key Findings	References
*C. difficile* infection (CDI)	Cohort study	Previous appendectomy history may worsen the severity of CDI.	[142]
CDI	Retrospective cohort study of 105,911 patients	CDI is infrequent following appendectomy for pediatric appendicitis but was more common in patients who took Ceftriaxone and Metronidazole.	[143]
CDI	Systematic review and meta-analysis	Appendectomy is not associated with an increased risk of severe CDI or recurrence (Odds ratio OR 1.03 (95% CI 0.6–1.78, *p* = 0.92)).	[144]
CDI	A multivariate logistic regression	The elderly, females, and those with sepsis and open surgery are at higher risk for developing CDI after an appendectomy.	[145]
*C. difficile* associated diarrhea (CDAD)	Retrospective cohort study of 12,039 patients	Cumulative CDAD rate was 2.3% after appendectomy with a median onset of 76 days. CDAD rate was significantly more common within 1 year following an appendectomy (37%)	[146]

**Table 5:** Recent research on the impacts of appendectomies on neurological disorders.

Disease Focus	Methodology	Key Findings	References
PD	Animal model study examining the impact of appendectomies on the gut–brain axis and neuroinflammation	Appendectomy in mice led to changes in GM and increased markers of neuroinflammation, suggesting a potential link to PD.	[155]
PD	A meta-analysis	No significant difference was observed in the emergence of PD between patients with or without appendectomy.	[156]
PD	A systematic review and meta-analysis	Appendectomy did not significantly affect PD risk (*p* = 0.789) or delay its onset (*p* = 0.083).	[157]
PD	Meta-analysis using 262 PD patients	Appendectomy alters the clinical expression of late-onset PD, with early cognitive impairment, and poorer functional independence seen with anti-parkinson’s medication.	[158]
PD	Prospective cohort study	There was no association between appendectomy and PD risk in men and women.	[149]
Alzheimer’s disease (AD)	Population-based cohort study	Patients developing appendicitis (0.6% vs. 0.1%, *p* = 0.005) and those receiving appendectomies (0.4% vs. 0.1%, *p* = 0.003) had higher rates of AD than the controls.	[159]
Multiple sclerosis (MS) and neuromyelitis optica spectrum disorder (NMOSD)	A statistical analysis based on 49 MS, 71 NMOSD, and 880 controls	MS is positively associated with appendectomy. Appendectomy rates were 18.37% in the MS group and 5.6% in the NMOSD group.	[160]
Mental disorders	A population-based cohort study of 1,937,488 people	Inidividuals over 14 who had previously recieved appendectomies had a 19% increased risk of depressive disorder, 27% increased risk of bipolar affective disorder, and a 20% increased risk of an anxiety disorder compared to the controls without childhood appendectomy	[161]
Sickle cell disease (SCA)	Retrospective review	There was no clear association between appendectomy and SCA	[162]

**Table 6:** Recent research on the impacts of appendectomy on heart disease, type 2 diabetes, and other complications.

Disease Focus	Methodology	Key Findings	References
Diverticular disease	German cohort study	There is a significant association between appendicitis and subsequent diverticular disease (HR: 1.76; 95% CI: 1.57–1.97) across all age groups.	[184]
Ischaemic heart disease (IHD)	Mendelian randomization (MR) study methods and meta-analysis	Genetically expected appendectomy could be a risk factor for the progress of IHD (OR: 1.128, 95% CI: 1.067–1.193, *p* = 2.459 × 10^−5^).	[170]
Viral myocarditis	Cohort study	Appendectomy alleviates coxsackievirus b3-induced viral myocarditis. The appendix modifies cardiac infection and inflammation by regulating IL-10^+^ Treg response.	[185]
Type 2 diabetes	A population-based cohort study	Appendectomy is associated with an increased risk of type 2 diabetes, particularly when performed before 30 years of age.	[175]
Mortality	Analyzed data on appendectomy deaths in Australia from 2006 to 2017	Mortality rate was very low. The overall mortality rate was 0.02%, with 0.01% in the young (age <65 years) and 0.20% in older people.	[186]
Mortality	Retrospective, cohort study of 65,625 subjects	Laparoscopic appendectomy (LA) was associated with 30-day mortality at 0.74 per 100 appendectomies.	[187]
Morbidity and mortality	A retrospective single-center study of 532 subjects	Postoperative morbidity and mortality were 14.4% and 0%, respectively.	[188]
Pregnancy rate	A single-center retrospective data analysis	Open appendectomy was safe in pregnancy, but increased cesarean deliveries.	[181]
Pregnancy rate	Retrospective cohort study of 1624 patients	LA was associated with a higher rate of preterm labor, preterm delivery, or abortion in the second (OR, 3.37; 95% CI, 1.76–6.47; and *p* < 0.001) and third trimesters (OR, 2.57; 95% CI, 1.15–5.70; and *p* = 0.021)	[189]
Pregnancy outcomes	A prospective cohort study	Appendectomy during pregnancy is associated with a low risk of perforation, preterm birth, and other complications.	[190]
Pregnancy outcomes	Data analysis of 2941 pregnant women	LA was feasible and safe during pregnancy	[191]
Rheumatoid arthritis (RA)	A population-based cohort study of 14,995 participants	No significant relationship between appendectomy and RA was found.	[192]
Systemic lupus erythematosus (SLE)	A nationwide cohort study	Women aged ≤49 years who underwent appendectomy had a 2.27-fold higher risk of SLE than the corresponding controls (adjusted HR = 2.27, 95% CI = 1.62–3.19).	[193]
Postoperative sepsis	Machine learning algorithms	Out of 223,214 identified appendectomies, 2143 (0.96%) were indicated as having postoperative sepsis.	[194]

## Data Availability

The dataset is available from the corresponding author upon reasonable request.
