# Correction: Islam et al. Exploring the Immunological Role of the Microbial Composition of the Appendix and the Associated Risks of Appendectomies. *J. Pers. Med.* 2025, *15*, 112

**DOI:** 10.3390/jpm16110573

**Published:** 2026-11-06

**Authors:** Tarequl Islam, Md Shahjalal Sagor, Noshin Tabassum Tamanna, Md Kamrul Islam Bappy, Danishuddin, Md Azizul Haque, Maximilian Lackner

**Affiliations:** 1 Department of Microbiology, Noakhali Science and Technology University, Noakhali 3814, Bangladesh; tarequembg@gmail.com (T.I.); kamrul0509@student.nstu.edu.bd (M.K.I.B.); 2 Department of Microbiology, Jagannath University, Dhaka 1100, Bangladesh; sagor@squaregroup.com; 3 Department of Pharmacy, Noakhali Science and Technology University, Noakhali 3814, Bangladesh; tamannanoshin@gmail.com; 4 Department of Biotechnology, Yeungnam University, Gyeongsan 38541, Republic of Korea; danish23@yu.ac.kr; 5 Department of Industrial Engineering, University of Applied Sciences Technikum Wien, Hoechstaedtplatz 6, 1200 Vienna, Austria

## 1. Text Correction

There were errors in the original publication [1]. Some references were not directly relevant to the statements they were cited for. These sentences have been revised.

Corrections have been made to the following sentences:

4.1. Immunological Roles, Paragraph 2, Sentence 3

Such immune training may help the body tolerate harmless antigens, and loss of this tolerance may lead to allergies and autoimmune diseases (ADs) [58,59].

4.2. Interaction with Immune System, Paragraph 3, Sentence 3

Additionally, appendix-associated microbes may aid in restoring the GM following antibiotic use or infections [17].

5.1. Gut Dysbiosis, Paragraph 2, Sentences 2–4

A cross-sectional study comparing individuals with and without a history of appendectomy (HwA and HwoA, respectively) revealed key differences in their gut microbial compositions; HwA individuals exhibited reduced microbial diversity, with lower abundance of beneficial SCFA-producing microbes such as *Roseburia*, *Barnesiella*, *Butyricicoccus*, *Odoribacter*, and *Butyricimonas* [19].

5.2. Risk of Colorectal Cancer (CRC), Paragraph 2, Sentence 3

However, an appendectomy remains the most frequently performed emergency surgical procedure, and recent evidence highlights its potential link to increased CRC risk [52,94,95,99].

5.2. Risk of Colorectal Cancer (CRC), Paragraph 3, Sentences 10–11

Altered GM may increase oxidative stress, which is linked to cancer development. While oxidative stress is a natural consequence of cellular metabolism, dysbiosis can exacerbate it, potentially contributing to carcinogenesis [104,109–111].

5.3. Risk of Inflammatory Bowel Diseases (IBDs), Paragraph 1, Sentences 1–3

Gut dysbiosis and alterations in microbial metabolic networks are closely associated with the pathogenesis of IBD [122]. Appendectomy has been associated with a reduced risk of UC, although its relationship with IBD remains complex, with an increased risk of CD reported in some epidemiological studies [65,100].

5.3. Risk of Inflammatory Bowel Diseases (IBDs), Paragraph 2, Sentence 7

Although these studies show a higher risk of CD after appendectomy, it remains unclear whether the surgery itself is the cause. An early international case–control study found that appendectomy was more common in patients with CD than in controls, but the study suggested that some of these operations may have been related to CD itself [129].

5.6. Risk of Amyotrophic Lateral Sclerosis (ALS), Paragraph 1, Sentences 6–7

These two sentences have been removed: Men are slightly more likely than women to develop ALS, though this gender difference decreases with age. Environmental factors, such as exposure to air pollution and certain toxins, may also play a role in increasing ALS risk [170,171].

6. Microbe-Based Therapies, Paragraph 2, Sentence 5

Extensive antibiotic use can disrupt the gut microbiota and contribute to the emergence of antibiotic-resistant pathogens [196].

## 2. Reference Correction

References 59, 81, 92, 93, 105, 170, 171, 204, and 205 have been removed from the original publication reference list. With this correction, the order of some references has been adjusted accordingly.

59. Lajqi, T.; Köstlin-Gille, N.; Bauer, R.; Zarogiannis, S.G.; Lajqi, E.; Ajeti, V.; Dietz, S.; Kranig, S.A.; Rühle, J.; Demaj, A.; et al. Training vs. Tolerance: The Yin/Yang of the Innate Immune System. *Biomedicines* **2023**, *11*, 766.

81. Smith, H.F.; Parker, W.; Kotzé, S.H.; Laurin, M. Multiple independent appearances of the cecal appendix in mammalian evolution and an investigation of related ecological and anatomical factors. *C. R. Pale.* **2013**, *12*, 339–354.

92. Rankin, L.C.; Girard-Madoux, M.J.H.; Seillet, C.; Mielke, L.A.; Kerdiles, Y.; Fenis, A.; Wieduwild, E.; Putoczki, T.; Mondot, S.; Lantz, O.; et al. Complementarity and redundancy of IL-22-producing innate lymphoid cells. *Nat. Immunol.* **2016**, *17*, 179–186.

93. Bonanni, P.; Grazzini, M.; Niccolai, G.; Paolini, D.; Varone, O.; Bartoloni, A.; Bartalesi, F.; Santini, M.G.; Baretti, S.; Bonito, C.; et al. Recommended vaccinations for asplenic and hyposplenic adult patients. *Hum. Vaccin. Immunother.* **2017**, *13*, 359–368.

105. Rotem-Kohavi, N.; Keane, J.; Clarke, G.; Dinan, T.G.; Cryan, J.F.; McCarthy, F.P.; Oberlander, T.F.; O’Mahony, S.M. The microbiome-gut-brain axis: A new window to view the impact of prenatal stress on early neurodevelopment. In *Prenatal Stress and Child Development*; Springer: Berlin/Heidelberg, Germany, 2021; pp. 165–191.

170. Zarei, S.; Carr, K.; Reiley, L.; Diaz, K.; Guerra, O.; Altamirano, P.F.; Pagani, W.; Lodin, D.; Orozco, G.; Chinea, A. A comprehensive review of amyotrophic lateral sclerosis. *Surg. Neurol. Int.* **2015**, *6*, 171.

171. Bradford, D.; Rodgers, K.E. Advancements and challenges in amyotrophic lateral sclerosis. *Front. Neurosci.* **2024**, *18*, 1401706. 

204. Islam, T.; Tamanna, N.T.; Sagor, M.S.; Zaki, R.M.; Rabbee, M.F.; Lackner, M. Antimicrobial Peptides: A Promising Solution to the Rising Threat of Antibiotic Resistance. *Pharmaceutics* **2024**, *16*, 1542.

205. Islam, T.; Danishuddin Tamanna, N.T.; Matin, M.N.; Barai, H.R.; Haque, M.A. Resistance Mechanisms of Plant Pathogenic Fungi to Fungicide, Environmental Impacts of Fungicides, and Sustainable Solutions. *Plants* **2024**, *13*, 2737.

The authors state that the scientific conclusions are unaffected. This correction was approved by the Academic Editor. The original publication has also been updated.
